# Vitamin D Exposure and Ovarian Cancer Risk and Prognosis

**DOI:** 10.3390/ijerph17041168

**Published:** 2020-02-12

**Authors:** Kevin L’Espérance, Geetanjali D. Datta, Samia Qureshi, Anita Koushik

**Affiliations:** 1 Department of Social and Preventive Medicine, Université de Montréal, Montreal, QC H2X 0A9, Canada; kevin.lesperance@umontreal.ca (K.L.); geetanjali.datta@umontreal.ca (G.D.D.); 2 Université de Montréal Hospital Research Centre (CRCHUM), Montreal, QC H2K 1H2, Canada; samia.qureshi@mail.mcgill.ca

**Keywords:** vitamin D, sun exposure, diet, 25(OH)D, ovarian cancer, risk, incidence, survival

## Abstract

Given the poor prognosis of ovarian cancer and limited population-level strategies for early detection and long-term treatment success, knowledge of modifiable risk factors for prevention and improved prognosis is important. Vitamin D has received wide scientific interest in cancer research as having the potential to be one such factor. We carried out a systematic narrative review of the literature on vitamin D and ovarian cancer risk and survival. We included 17 case-control and cohort studies on ovarian cancer incidence. Five analyses were of sun exposure, among which three reported an inverse association. Of 11 analyses of dietary vitamin D, two reported an inverse association. Among five studies of 25(OH)D levels, an inverse association was reported in two. Across all studies the findings were inconsistent, but some recent studies have suggested that vitamin D exposure at earlier ages may be important. Only three studies examining vitamin D exposure in relation to survival among ovarian cancer survivors were identified and the findings were inconsistent. The evidence to date supports a null influence of vitamin D on both ovarian cancer risk and survival. Future research should ensure that exposure assessment captures vitamin D exposure from all sources and for the etiologically or prognostically pertinent period.

## 1. Introduction

Worldwide, ovarian cancer is the eighth most frequently diagnosed cancer among women, but incidence rates vary geographically, with the highest rates in Europe and North America, and the lowest in Africa and Asia [1]. Survival is very poor, with age-standardized five-year net survival ranging from 36% to 46% in high income countries [1]. The poor prognosis is a consequence of the fact that most ovarian cancers are aggressive such that, at diagnosis, the disease has spread beyond the pelvis reducing the chances of long-term treatment success. Prevention is of critical importance in controlling ovarian cancer, but few modifiable risk factors are known. Similarly, the only factors known to influence survival are clinical or biological in nature, such as age at diagnosis and stage, grade and histology of the cancer, and thus are not modifiable.

Vitamin D has received wide scientific interest in cancer prevention research [2,3]. The main source of vitamin D in the body is cutaneous exposure to ultraviolet-B (UVB) radiation from the sun that catalyzes the conversion of 7-dehydrocholesterol to vitamin D_3_ (cholecalciferol) [4,5]. Diet also constitutes a source of both vitamin D_2_ (ergocalciferol) and vitamin D_3_ [4,5] through natural sources (e.g., fish, eggs), fortified foods (e.g., milk, breakfast cereals) and supplements [6]. Vitamins D_2_ and D_3_ are then converted in the liver to 25-hydroxyvitamin D (25(OH)D: calcidiol), the circulating form of vitamin D, and subsequently transformed in the kidneys to 1,25-dihydroxyvitamin D (1,25(OH)_2_D: calcitriol), the hormonally active form [4]. 

Experimental research in ovarian cancer cell lines and mouse and human xenograft models has provided strong evidence that vitamin D can decrease proliferation, increase apoptosis and suppress tumor progression [7,8,9,10,11] [9,11,12]. However, epidemiological research on vitamin D exposure and ovarian cancer has not been conclusive [13,14,15]. Study design and the measure of vitamin D examined have varied across studies. For instance, the ecologic studies included in a 2010 systematic review [13] used measures of sun exposure to represent vitamin D; however, these studies are limited in having used a group-level measure of ovarian cancer incidence. Among the 10 individual-level studies in that review [13], which used case-control and cohort designs, all but 2 examined dietary vitamin D only. Circulating 25(OH)D measures total vitamin D exposure and thus represent an improvement over the study of diet or sun exposure only. In a pooled analysis of seven cohort studies, a null association between 25(OH)D and ovarian cancer risk was reported [14]. However, in a subsequent 2011 meta-analysis of the same seven studies plus an additional three, a suggestive but statistically non-significant inverse association was observed [15]. Furthermore, in a Mendelian randomization study, genetically lowered 25(OH)D level was associated with a statistically significant higher ovarian cancer risk, supporting a role of vitamin D on ovarian cancer [16]. A more recent Mendelian randomization study did not observe an association between genetically determined 25(OH)D and ovarian cancer, but the sample size and thus statistical power was limited [17]. Studies investigating genetic variants of the vitamin D receptor, which mediates the activity of 1,25(OH)D [18], have suggested that vitamin D influences ovarian risk as well as survival [19,20,21,22,23,24,25,26]. 

Overall, there is biologic plausibility for a relation between vitamin D and ovarian cancer, which is supported by experimental research, but the epidemiological evidence remains unclear. Since the three previous reviews [13,14,15], there have been six additional studies published on vitamin D and ovarian cancer risk [27,28,29,30,31,32], as well as three studies on the relation between vitamin D and ovarian cancer survival [33,34,35]. We carried out a systematic narrative review of the literature on vitamin D and ovarian cancer risk and survival to summarize the literature to date. 

## 2. Materials and Methods

### 2.1. Data sources and Search Strategy

This systematic narrative review was guided by the Preferred Reporting Items for Systematic Reviews and Meta-Analysis (PRISMA) statement [36]. Searches on MEDLINE and EMBASE were conducted up to December 2019. For studies of incidence, we searched [ovarian cancer OR ovarian carcinoma* OR ovarian neoplasm*] AND [risk OR incidence] AND [vitamin D OR *calciferol OR sunlight OR sun OR latitude OR milk OR dairy OR ultraviolet OR UVB OR irradiance]. For studies of survival, we searched [ovarian cancer OR ovarian carcinoma* OR ovarian neoplasm*] AND [survival OR mortality OR prognosis] AND [vitamin D OR *calciferol OR sunlight OR sun OR latitude OR milk OR dairy OR ultraviolet OR UVB OR irradiance]. The reference lists of the identified articles were also searched to identify additional relevant studies. 

### 2.2. Selection Criteria

Titles and abstracts were reviewed for eligibility by two authors (A.K. and G.D.D.). In the case of a disagreement, a third reviewer (K.L.) assessed the title and abstract. Studies that met the following eligibility criteria were included in this review: (1) the exposure of interest was vitamin D or sun exposure (as a proxy for vitamin D) and the outcome of interest was ovarian cancer incidence or survival; (2) an estimate of relative risk (RR) was reported; and (3) the study was published in English or French. Given the unique set of potential biases with ecologic studies [37], we restricted our review to studies in which ovarian cancer incidence or survival was measured at the individual level. Reviews and meta-analyses were excluded except to identify pertinent references; however, pooled analyses were included. 

### 2.3. Data Extraction and Quality Assessment

Through full-text review, the following information was extracted from each study by K.L. and S.Q.: study design, location, study population, sample size, type of vitamin D exposure measured, time period for which vitamin D exposure was measured, RR estimate for the highest vs lowest exposure groups and the covariates included in models. When multiple models were reported, the most adjusted RR was extracted. In studies of diet, we extracted the RR estimate for vitamin D from diet plus supplements over diet only when both were available. The methodologic quality of the included studies was assessed using the Newcastle-Ottawa scale. We considered a score of 7 or above as high quality, from 4 to 6 as intermediate quality and from 0 to 3 as poor quality. The data extraction and assessment of quality was subsequently reviewed by A.K. and G.D.D.

### 2.4. Analysis

Due to the heterogeneity in type of vitamin D exposure measure across studies, a quantitative meta-analysis was not carried out. The results of this review are summarized narratively and in tables according to outcome (i.e., ovarian cancer incidence and survival) and type of vitamin D exposure measure (i.e., sun exposure, dietary intake and plasma levels), with tables organized according to year of publication and study design.

## 3. Results

### 3.1. Study Selection

**Figure 1:**
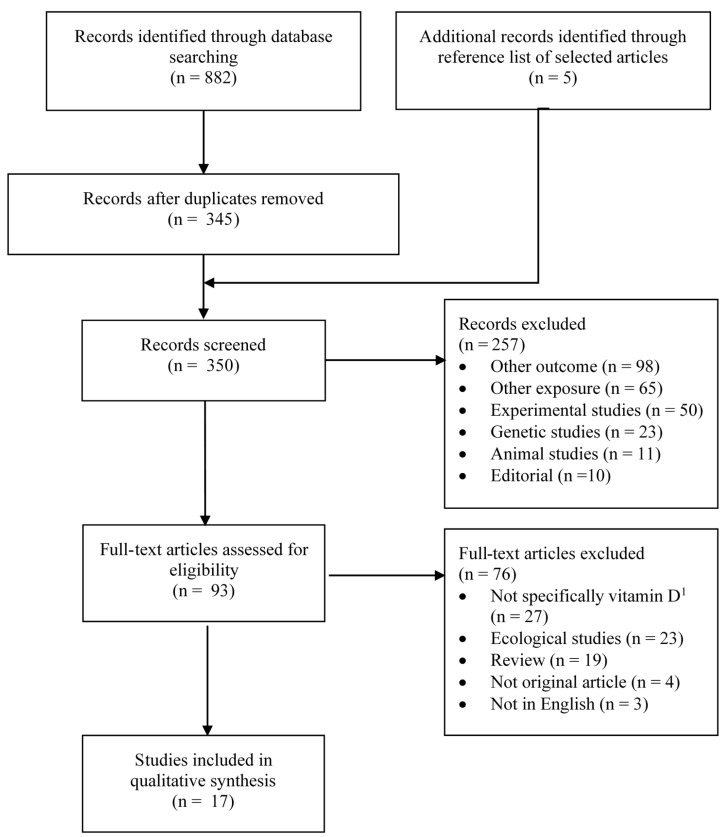
Flow diagram of search and screening process for vitamin D exposure and ovarian cancer incidence. n = number; ^1^ e.g., exposure was milk intake, fatty fish intake, serum alkaline phosphatase.

**Figure 2:**
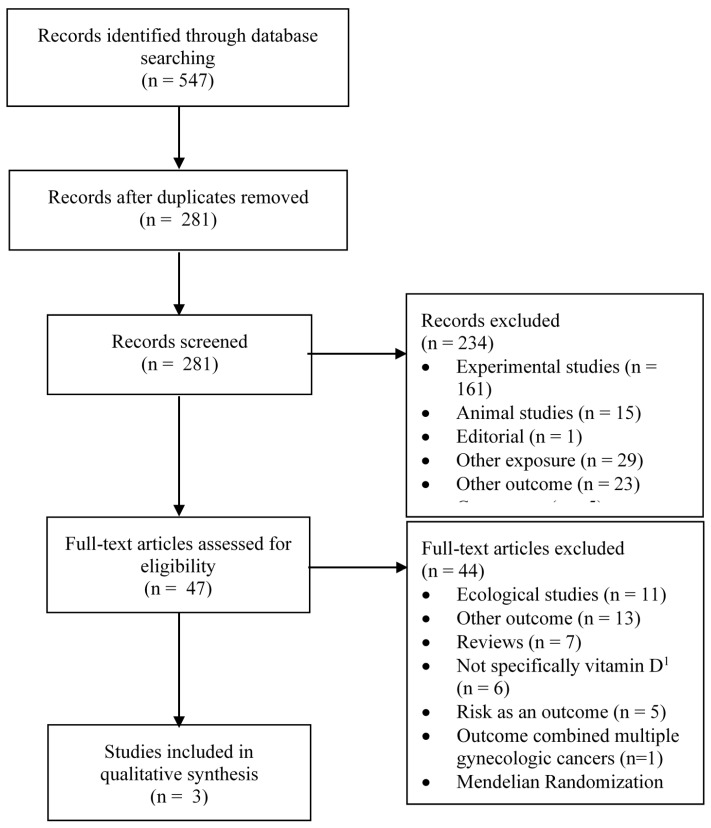
Flow diagram of search and screening process for vitamin D exposure and ovarian cancer survival. n = number; ^1^ e.g., exposure was dairy product intake, multivitamin use.

Figure 1 and Figure 2 summarize the identification, screening and selection of studies on ovarian cancer incidence and survival, respectively. A total of 350 independent articles on ovarian cancer incidences were found, among which 93 were judged to be eligible for full-text review (Figure 1). Seventeen articles met our inclusion criteria [14,27,28,29,30,31,32,38,39,40,41,42,43,44,45,46,47], of which nine had been included in a previous systematic review [13] and four in a previous meta-analysis [15]. Two articles reported the results of pooled analyses [14,41], which included some individual studies on vitamin D exposure and ovarian cancer that had not been previously published. Of the 17 eligible studies, vitamin D was assessed according to sun exposure in four studies [27,28,29,31], dietary intake in 10 studies [28,29,30,39,40,41,42,43,44,45] and circulating 25(OH)D levels in five studies [14,32,38,46,47]. For ovarian cancer survival, a total of 281 independent articles were found, of which 47 were judged to be eligible for full-text review and three were retained (Figure 2) [33,34,35]. 

.

### 3.2. Studies of Sun Exposure and Ovarian Cancer Incidence

Table 1 summarizes the studies that utilized sun exposure as a proxy for vitamin D exposure, including three case-control studies [27,28,31] and one study that reported results from two separate cohorts [29]. The specific measure used across the studies was ambient UV radiation at the location of residence, except for one where participants reported the number of daylight hours spent outdoors [28]. All studies were considered to be of intermediate to high methodological quality. Of the case-control studies, two reported a statistically significant inverse relationship with risk [27,28], among which one measured recent sun exposure [28], while in the other sun exposure was for the period from age 5 to study participation [27]. In contrast, a third study measuring mean levels of erythemal exposure (EE) from age 25 to study participation as an indicator of the potential biological damage from UV radiation reported no association between exposure and risk [31]. In the cohort study, which included data from the Nurses’ Health Study (NHS) and the Nurses’ Health Study II (NHSII) [29], baseline sun exposure was not associated with risk in the NHS but a statistically significant inverse association was reported in the NHSII. Associations with estimates of sun exposure at birth, age 15, age 30 and averaged from baseline to end of follow-up also showed an inverse association in the NHSII and no association in the NHS [29]. 

### 3.3. Studies of Dietary Vitamin D and Ovarian Cancer Incidence

Table 2 summarizes the six case-control studies [28,30,39,40,42,45], three cohort studies [29,43,44] and one pooled analysis of multiple cohorts [41] that investigated dietary intake of vitamin D in relation to ovarian cancer risk. The case-control studies were all of intermediate to high methodological quality. Diet was generally measured for the period one to two years prior to study participation. Two reports [30,40] utilized the same study population, with one [30] representing a larger sample from continued recruitment. Among these case-control studies, a moderate to strong inverse association was reported in two [39,45], the association was null in three [28,30,40] and the association was suggestive of an increased risk in another [42]. The cohort studies were also of intermediate to high methodological quality [29,43,44]. Diet was measured at baseline among women without ovarian cancer. Cut-points for the highest level of exposure were generally greater than 300 IU/day across studies, while intake in the reference categories were generally less than 200 IU/d. In the study including the NHS and NHSII, the measure of dietary vitamin D took into account supplements and the cumulative average from baseline to end of follow-up was assessed [29]. The reported associations among the cohort studies were generally null, although a slightly increased RR that was not statistically significant was reported in one study [44]. A null association was reported in a pooled analysis of seven cohort studies [41], which included four of the individual cohorts in this review [29,43,44]; the others did not publish their individual study results. 

### 3.4. Studies of Circulating 25(OH)D and Ovarian Cancer Incidence

Table 3 summarizes the studies that examined circulating 25(OH)D in relation to ovarian cancer risk [14,32,38,46,47], all of which were case-control studies nested in established prospective cohorts and were of high methodological quality. Three of the eligible studies were pooled analyses of multiple cohorts [14,38,47]. All studies used blood samples from a single time point at the cohort baseline, except one in which the sample used was that which was collected closest to diagnosis for cases or closest to selection for controls, within 10 years [46]. Circulating 25(OH)D was determined via radioimmunoassay. In a study of 224 cases pooled from the NHS, NHSII and Women’s Health Study [47], the observed RR for highest vs lowest 25(OH)D level was 0.83 and not statistically significant. The reported RR comparing the highest to lowest levels was closer to null in another pooled analysis that included 316 cases from the New York University Women’s Health Study (NYUWHS) and the Umea Northern Sweden Health and Disease Study [38]. Conversely, the results from two separate analyses from the Finnish Maternity Cohort suggested inverse associations between 25(OH)D levels and ovarian cancer incidence when comparing the highest vs lowest levels of exposure [32,46]. In one of the studies, the RR was stronger when cases with blood samples collected within one to three years of diagnosis were excluded [32]. In the other, a null association was observed when examining blood samples for cases and controls collected in opposite seasons [46]. In a pooled analysis of seven cohort studies [14] that included two studies that were in the previously mentioned pooled analyses (i.e., the NHS and the NYUWHS), a null association was observed overall, though a possible inverse association was suggested among overweight and obese women. Other than the NHS and NYUWHS, which were included in other pooled analyses [38,47], none of the individual studies had published their findings on circulating 25(OH)D and ovarian cancer risk.

### 3.5. Studies of Vitamin D Exposure and Ovarian Cancer Survival

The relation between vitamin D exposure and survival among women diagnosed with ovarian cancer was examined in three cohort studies conducted in Norway [33], Poland [34] and Australia [35] (Table 4). Methodological quality was considered poor in one study but high in the two others. In one study, the ultraviolet index based on region of residence and season at diagnosis was used as a proxy for vitamin D [33], and hazard ratios of mortality by region indicated no association. Two studies used circulating 25(OH)D as the exposure [34,35], and high vs low pre-surgical 25(OH)D levels were associated with a significantly higher five-year survival in one of them [34]. In the other, circulating 25(OH)D at diagnosis was also significantly inversely associated with survival [35]. However, in that study, when 25(OH)D levels after treatment was analyzed, the inverse association was attenuated [35]. 

## 4. Discussion

To the best of our knowledge, the current review is the first to summarize studies of vitamin D exposure and survival among ovarian cancer survivors. Only three such studies were identified, and their findings are inconsistent. For studies of incidence, we identified and included six studies that had not been included in previous reviews [27,28,29,30,31,32]. Of these, four reported on sun exposure [27,28,29,31], among which two also reported on dietary vitamin D along with one other study [28,29,30], while another reported on plasma vitamin D [32]. None of the previous reviews had included studies on sun exposure where ovarian cancer was measured at the individual level. Among the five analyses reviewed [27,28,29,31], three reported statistically significant reduced ovarian cancer risks with higher sun exposure [27,28,29]. Among the three studies of diet not previously reviewed [28,29,30], the reported RRs were null. Finally, among the study of 25(OH)D not previously reviewed [32], a non-significant inverse association was suggested. Across all 17 studies included in this review, the evidence was not consistent. However, the more recent studies had a tendency to report an inverse association between vitamin D exposure and ovarian cancer risk.

An inverse association between vitamin D and ovarian cancer risk was suggested primarily among the studies of sun exposure [27,28,29]. Although these studies were at the individual level, having used case-control and cohort designs, the measure of sun exposure was ecologic in nature, with a value for a defined geographical area having been assigned to all individuals residing in that area. Individual factors that may affect individual exposure, such as time spent outside and sun protective behaviors (e.g., sun screen use), were not accounted for. As any consequent misclassification of exposure would be similar among comparison groups, resulting in attenuated RR estimates, the relatively strong inverse associations observed in three of the five analyses may reflect the fact that sun exposure is the most important source of vitamin D in the human body [48]. Our observation that only two of the 11 analyses on dietary vitamin D reported inverse associations with risk may reflect exposure misclassification, but may also be due to the fact that dietary sources of vitamin D contribute less to overall vitamin D in the body than sun exposure. It is important to note, however, that dietary sources and supplemental intake are important among people with low sun exposure [49].

Among the studies of 25(OH)D levels, which provide a complete measure of vitamin D exposure from all sources, three of the five studies reported null associations. A common aspect of the two studies that reported nonsignificant inverse associations with risk is that they were both nested in the Finnish Maternity Cohort [32,46]. This cohort includes pregnant women, with blood samples drawn during the first trimester. Unlike the other cohort studies that have examined 25(OH)D in relation to ovarian cancer risk, women in the Finnish Maternity Cohort are not postmenopausal at baseline and thus the average age of the study participants is lower. In general, cancers are known to develop over long periods [50]. For ovarian cancer, the protective effects of pregnancy and oral contraceptive use [51,52,53], the only two established risk factors for which exposure usually occurs in early adulthood, suggest that this period may be important in ovarian cancer development. Thus, the inverse association with 25(OH)D suggested in the two analyses from the Finnish Maternity Cohort may reflect that the measurement of vitamin D better captured the etiologically relevant period for ovarian cancer development.

Timing of exposure measurement may also explain the inverse associations with risk observed for some studies of sun exposure. For instance, in the Australian study [27], sun exposure was represented by average lifetime exposure from age 5, thus having possibly covered the etiologically relevant period. In the other study reporting a reduced risk with sun exposure [28], which was conducted among African American women, a time period of exposure was not specified; rather, participants reported their average weekly time spent outdoors during the summer months. The exposure measure may have captured the relevant period only if time spent outdoors did not vary greatly throughout life. The conflicting results with UVB flux at baseline in the NHS vs NHSII [29] may be explained by timing of exposure given that, at baseline, participants in the NHSII were on average in their early 30s as compared to their early 50s in the NHS. However, results from analyses for UVB flux at birth, age 15, age 30 and averaged from baseline to the end of the follow-up also demonstrated the same differences between the two cohorts. Secular trends in parity, oral contraceptive use and other potential risk factors such as obesity, smoking and hormone therapy may contribute to these differences [54].

Exposure assessment that captures all vitamin D sources for the etiologically pertinent period will be important to better understand the influence of vitamin D on ovarian cancer incidence. There is growing recognition of the role early life exposures play on later cancer risk [55]. If vitamin D exposure plays a more important role on ovarian cancer risk during early life, future studies based on new birth cohorts or cohorts of children, adolescents or young adults will be informative. However, given the long induction period of ovarian and other cancers, and the resources required to carry out such studies [55], it will be many years before such data are available. With respect to capturing all vitamin D sources, the best available biomarker is plasma 25(OH)D level [56]. However, the cost of its measurement can be prohibitive in epidemiologic studies. Indeed, the studies of 25(OH)D included in our review were limited in power due to relatively small sample sizes. A proposed cost-efficient option to obtain a measure of total vitamin D exposure from all sources is to use validated regression models that predict 25(OH)D levels from self-reported lifestyle, environment and personal characteristics that influence levels and that are more easily obtained in large studies [57,58,59]. In the study that included the NHS and NHSII [29], such an approach was used in which a higher cumulative average predicted 25(OH)D was associated with a slightly increased risk in the NHS and a lower risk in the NHSII, both of borderline statistical significance. It may be possible to apply such models to data pertaining to early life in order to estimate predicted vitamin D levels for different periods throughout the life course.

For the role of vitamin D on ovarian cancer survival, more research is needed, given that very few studies have been conducted and that vitamin D was measured around the time of diagnosis in most studies. As is the case for studies of incidence, it is of importance to measure all vitamin D sources and to capture the relevant timing of exposure. Indeed, the majority of women first diagnosed with ovarian cancer achieve remission [60], but about 80% will face a recurrence of disease [61]. In order to delay recurrence and improve survival, it is necessary to understand the role of vitamin D, if any, at a moment that intervention can take place, which would be when remission is established. Moreover, the 25(OH)D levels in blood collected around the time of diagnosis or treatment may be affected by the cancer or the treatment. Thus, attention to timing of vitamin D measurement is important in survivorship studies.

## 5. Conclusions

Overall, the current evidence supports a null influence of vitamin D on both ovarian cancer risk and ovarian cancer survival. However, an inverse association suggested in some recent studies where the measure of vitamin D was for earlier ages suggests that some of the older studies may have not captured a complete measure of vitamin D exposure and/or may have missed the etiologically relevant period in which vitamin D exposure is important in cancer development. Future etiologic studies would benefit from including measures of total vitamin D, possibly via the use of prediction models, and assessing women of younger ages. Future survival studies could benefit from assessing vitamin D exposure at remission as a potential intervention point in improving outcomes. While the early promise of vitamin D as a modifiable cancer preventive factor has not yet been realized, more evidence is necessary in order to rule out a targeted role for vitamin D in ovarian cancer etiology and survival.

## Figures and Tables

**Table 1:** Studies of sun exposure in relation to ovarian cancer risk (incidence).

Author, Year of Publication [Reference]	Study Location	Study Design	Recruitment Period or Cohort Follow-up Years	No. Cases	No. Controls or Cohort Size	Measure of Sun Exposure	Timing of the Exposure Measurement	RR (95% CI) for Highest vs Lowest Exposure	Adjustment Variables	Study Quality ^1^
Bodelon, 2012 [31]	USA	Population-based case control	2002–2009	1334	1679	Mean erythemal exposure (EE) based on residential history	From age 25 to one year before study participation	0.97 (0.79–1.19) ^2^	Age, county of residence, calendar year, number of full-term pregnancies and duration of hormonal contraceptives.	8
Tran, 2012 [27]	Australia	Population-based case control	2002–2005	1500	1459	Average daily ambient ultraviolet radiation based on residential history	From age 5 to study participation	0.73 (0.57–0.95)	Age, state of residence, body mass index, ever breastfeeding, parity, use of hormonal contraceptive pills and family history of breast/ovarian cancer.	6
Qin, 2016 [28]	USA	Population-based case control	2010–2016	490	656	Daylight hours spent outdoors in summer months	Not specified	0.71 (0.51–0.99)	Age, region, total energy intake, education, parity, oral contraceptive use, menopausal status, tubal ligation, family history of breast/ovarian cancer, pigmentation, recreational physical activity, body mass index and total vitamin D intake.	7
Prescott, 2013 ^3^ [29]	USA	Prospective cohort	1976–2010	970	75,613 ^4^	Ultraviolet-B (UVB) flux based on latitude, altitude and cloud cover	Baseline	1.13 (0.96–1.33)	Age, duration of oral contraceptive use, number of pregnancies, tubal ligation, menopausal status, ever use of post-menopausal hormones and first-degree family history of ovarian cancer.	6
Prescott, 2013 ^3^ [29]	USA	Prospective cohort	1989–2011	255	102,904 ^4^	UVB flux based on latitude, altitude and cloud cover	Baseline	0,70 (0.53–0.93)	Age, duration of oral contraceptive use, number of pregnancies, tubal ligation, menopausal status, ever use of post-menopausal hormones and first-degree family history of ovarian cancer.	6

^1^ Score based on the Newcastle-Ottawa Scale; ^2^ The category of reference is an intermediate level of exposure; ^3^ This study reported on two cohort studies, the Nurses’ Health Study (NHS) and the Nurses’ Health Study II (NHSII); ^4^ Cohort size at mid-point of follow-up.

**Table 2:** Studies of dietary vitamin D in relation to ovarian cancer risk (incidence).

Author, Year of Publication [Reference]	Study Location	Study Design	Recruitment Period or Cohort Follow-up Years	No. Cases	No. Controls or Cohort Size	Type of dietary vitamin D Source Assessed	Timing of Diet Assessment	RR (95% CI) for Highest vs. Lowest Exposure	Adjustment Variables	Study Quality ^1^
Bidoli, 2001 [39]	Italy	Hospital-based case-control	1992–1999	1031	2411	Diet only	2 years prior to study participation	Quintile 5 vs. 1 0.7 (0.6–1.0)	Age, study center, year of interview, education, body mass index, parity, oral contraceptive use, occupational physical activity and energy intake.	6
Cramer, 2001 ^2^ [40]	USA	Population-based case-control	1992–1997	549	516	Diet only	1 year prior to study participation	>584 vs. ≤162 IU/d 0.99 (0.65–1.52)	Caloric intake, age, site, parity, body mass index, oral contraceptive use, family history of breast / prostate / ovarian cancer, tubal ligation, education, marital status and supplements.	6
Goodman, 2002 [42]	USA	Population-based case-control	1993–1999	558	607	Diet and supplement use	1 year prior to study participation	Quartile 4 vs. 1 1.49 (0.90–2.47)	Age, ethnicity, study center, education, use of oral contraceptives, parity, tubal ligation, energy intake, lactose intake and calcium intake.	8
Salazar-Martinez, 2002 [45]	Mexico	Hospital-based case-control	1995–1997	84	629	Diet only	Not specified	≥360 vs ≤214 IU/d 0.43 (0.23–0.80)	Age, total energy intake, number of live births, recent changes in weight, physical activity and diabetes.	6
Merritt, 2013 ^2^ [30]	USA	Population-based case-control	1993–2008	1909	1989	Diet and supplement use	1 year prior to study participation	>559.1 vs. <163.6 IU/d 0.93 (0.74–1.16)	Age, number of pregnancies, oral contraceptive use, tubal ligation, history of ovarian cancer in family, study center and phase, total energy intake.	7
Qin, 2016 [28]	USA	Population-based case-control	2010–2016	490	656	Diet and supplement use	1 year prior to study participation	≥524.0 vs. ≤130.8 IU/d 1.00 (0.65–1.54)	Age, region, total energy intake, education, parity, oral contraceptive use, menopausal status, tubal ligation, family history of breast / ovarian cancer, daylight hours spent outdoors in summer months, pigmentation, recreational physical activity, body mass index, other sugar intake excluding lactose, and total calcium and total lactose intake.	7
Kushi, 1999 [44]	USA	Prospective cohort	1986–1995	139	2,9083	Diet only	Baseline	>566 vs. <198.5 IU/d 1.37 (0.81–2.32)	Age, total energy intake, number of livebirths, age at menopause, family history of ovarian cancer in first-degree relatives, hysterectomy/ unilateral oophorectomy status, waist-to-hip ratio, level of physical activity, cigarette smoking and educational level.	7
Koralek, 2006 [43]	USA	Prospective cohort	1987–1998	146	3,1925	Diet and supplement use	Past year	Quartile 4 vs. 1 1.08 (0.63–1.87)	Total calcium, lactose, age, menopause type, parity, age at menarche, oral contraceptive use and post-menopausal hormone use at baseline.	7
Prescott, 2013 ^3^ [29]	USA	Prospective cohort	1980–2010	731	75,613 ^4^	Diet only	Cumulative average from baseline to end of follow-up	≥300 vs. <200 IU/d 0.96 (0.76–1.20)	Age, duration of oral contraceptive use, number of pregnancies, tubal ligation, menopausal status, ever use of post-menopausal hormones, first-degree family history of ovarian cancer, and total caloric intake.	6
Prescott, 2013 ^3^ [29]	USA	Prospective cohort	1991–2011 ^5^	200	10,2904 ^4^	Diet only	Cumulative average from baseline to end of follow-up	≥300 vs. <200 IU/d 1.03 (0.71–1.50)	Age, duration of oral contraceptive use, number of pregnancies, tubal ligation, menopausal status, ever use of post-menopausal hormones, first-degree family history of ovarian cancer, and caloric intake.	6
Genkinger, 2006 ^5^ [41]	Multiple	Pooled analysis of 7 prospective cohorts^2^	Study-specific follow-up	1296	40,8824	Diet and supplement use	Past year, for most studies	≥500 vs. <100 IU/d 1.12 (0.90–1.38)	Age at menarche, menopausal status, oral contraceptive use, hormone replacement therapy use, parity, smoking status, physical activity and energy intake.	N/A

^1^ Score based on the Newcastle-Ottawa Scale; ^2^ Cramer, 2001 and Merritt, 2013 represent the same study population; ^3^ This study reported on two cohort studies, the Nurses’ Health Study (NHS) and the Nurses’ Health Study II (NHSII); ^4^ Cohort size at mid-point of follow-up; ^5^ In this pooled analysis of 12 cohort studies, 7 had assessed total vitamin D intake; table entries on cohort size and number of cases refer strictly to the analysis of total vitamin D.

**Table 3:** Nested case-control studies of circulating 25(OHD) levels in relation to ovarian cancer risk (incidence).

Author, Year of Publication [Reference]	Study Location	Cohort Follow-up Years	No. Cases	No. Controls	Timing of Blood Draw for Vitamin D Measurement	RR (95% CI) for Highest vs. Lowest Exposure	Adjustment Variables	Study Quality ^1^
Tworoger, 2007 [47]	USA	Three cohorts pooled: NHS ^3^: 1989–2004 NHSII ^3^: 1996–2003 WHS ^3^: 1992–2004	224	603	Study baseline	Study-specific cut points ^2^: NHS/NHSII^3^: ≥81.1 vs. <51.4 nmol/L WHS ^3^: ≥69.1 vs. <43.4 nmol/L Pooled RR: 0.83 (0.49–1.39)	Matched for having intact ovaries at time of the case diagnosis, menopausal status at baseline and diagnosis, age, month, time of day and postmenopausal hormone use at blood draw, fasting status and day of luteal blood draw. Multivariable model adjusted for ever use of postmenopausal hormones, body mass index at blood draw, parity, lactose intake, duration of oral contraceptive use and interaction between duration of oral contraceptive use and body mass index at blood draw.	9
Arslan, 2009 [38]	USA, Sweden	Two cohorts pooled: NYUWHS ^3^: 1985-2005 NSHDS ^3^: 1985–2005	168	316	Baseline	Study-specific cut points: NYUWHS^3^: ≥57.8 vs. ≤36.7 nmol/L NSHDS^3^: ≥44.8 vs. ≤34.0 nmol/L Pooled RR: 1.09 (0.59–2.01)	Matched for cohort, age at entry and date of blood donation. Multivariable model adjusted for oral contraceptive use and parity.	9
Toriola, 2010a [46]	Finland	1983–2006	201 ^4^	398/198 ^5^	Closest blood donation within 10 years from diagnosis or enrollment	Same season: <26.4 vs. ≥53.1 nmol/L 1.8 (0.9-3.5) ^6^ Opposite season: <25.3 vs. ≥51.9 nmol/L 1.1 (0.6–2.2) ^6^	Matched for age at blood withdrawal, parity and index of blood sampling. Multivariable model adjusted for age at last full-term pregnancy and bench lag-time.	10
Toriola, 2010b [32]	Finland	1983–2007	168 ^7^	172	Cohort baseline, which was first pregnancy, and at least 1 year before cancer diagnosis	≥57.8 vs. <31.5 nmol/L 0.57 (0.26–1.24)	Matched for age at blood withdrawal, parity and index of blood sampling. Age at first full-term pregnancy and region of residence.	10
Zheng, 2010 ^8^ [14]	Multiple	Study-specific follow-up	516	770	Baseline	≥100 vs. 50-<75 nmol/L 1.11 (0.61–2.05)	Matched for age, month of blood collection, time of day of blood draw, fasting status, menopausal status and postmenopausal hormone use at blood draw. Multivariable model adjusted for race/ethnicity, study cohort, duration of oral contraceptive use and number of pregnancies.	N/A

^1^ Score based on the Newcastle-Ottawa Scale; ^2^ In the original article, 25(OH)D values were expressed in units of ng/mL; they have been converted to units of nmol/L in this table for consistency with the other studies; ^3^ NHS, Nurses’ Health Study; NHSII, Nurses’ Health Study II; WHS, Women’s Health Study; NYUWHS, New York University Women’s Health Study; NSHDS, Northern Sweden Health and Disease Study; ^4^ The 201 cases are a random sample of all cases from the cohort; ^5^ There were two control groups: 398 controls donated serum in the same season as case, 198 controls donated serum in the opposite seasons; ^6^ The RRs are for the comparison of the lowest vs highest exposure; ^7^ The cases in this study do not overlap with the cases in Toriola, 2010a; ^8^ Nested case: control study in 7 prospective cohorts: Cancer Prevention Study II Nutrition Cohort; Nurses’ Health Study; Nurses’ Health Study II; New York University Women’s Health Study; Multiethnic Cohort Study; Prostate, Lung, Colorectal, and Ovarian Cancer Screening Trial; and Shanghai Women’s Health Study.

**Table 4:** Cohort studies of vitamin D exposure in relation to ovarian cancer survival.

Author, Year of Publication [reference]	Study Location	Follow-Up Years	Cohort Size	No. of Outcomes	Measure of Vitamin D	Measure of Association with Survival	Adjustment Variables	Study Quality ^1^
Porojnicu, 2008 [33]	Norway	1964–2000	42,096	7112	Ultraviolet index based on residential region and season at diagnosis	RRs of death at 36 months were 1 for all region/season comparisons	Age, sex, birth cohort, stage of disease and UV index.	8
Walentowicz-Sadlecka, 2012 [34]	Poland	2005–2011	72	Not mentioned	Circulating 25(OH)D on day of before surgery	Overall survival at 5 years for high vs low 25(OH)D, 46.3% vs 25.8%, respectively	None	3
Webb, 2015 [35]	Australia	2002–2011	670 ^2^	435 with disease progression or death	Circulating 25(OH)D at diagnosis	RR (95% CI): 0.93 (0.88, 0.99) per 10 nmol/L	Age, state of residence, smoking status at diagnosis and body mass index.	8
Webb, 2015 [35]	Australia	2002–2011	279 ^2^	160 with disease progression or death	Circulating 25(OH)D after treatment (but before disease progression)	RR (95% CI): 0.97 (0.89, 1.06) per 10 nmol/L	Age, state of residence, smoking status at diagnosis and body mass index	8

^1^ Score based on the Newcastle-Ottawa Scale; ^2^ Women with blood drawn at diagnosis and after treatment were analyzed separately.
